# Plaie du tendon d'Achille: technique chirurgicale mini-invasive

**DOI:** 10.11604/pamj.2016.24.6.8550

**Published:** 2016-05-03

**Authors:** Hassane Zejjari, Khalid Rachid

**Affiliations:** 1 Service de Chirurgie Traumatologique et Orthopédique de l'Hôpital Militaire Moulay Ismail De Meknès, Maroc

**Keywords:** Tendon d'Achille, chirurgie mini-invasive, Maroc, Achilles tendon, minimally invasive surgery, Morocco

## Image en médecine

Le tendon calcanéen est le plus volumineux et le plus résistant tendon de l'organisme. C'est en 1883 que Pollailon a décrit la première intervention inaugurant l’ère chirurgicale des ruptures du tendon calcanéen. La réparation à « ciel ouvert » apparaît alors comme le plus sûr moyen d'assurer un contact solide des extrémités permettant une cicatrisation satisfaisante et rétablissant une longueur du tendon optimale d'un point de vue biomécanique. Nous rapportons une image illustrant une technique chirurgicale mini-invasive personnelle inspirée d'une étude de la littérature. Il s'agit d'un patient âgé de 24 ans victime d'une plaie du tendon d'Achille par arme blanche. La technique consiste à réaliser une suture en cadre permettant d'affronter les deux bouts tendineux. Deux fils non résorbables sont introduits de façon horizontale de chaque côté de la rupture en percutané puis ils sont extrais à travers la plaie horizontal grâce à un crochet mousse. Les fils sont serrés la cheville étant en équin. Le bon affrontement des deux bouts tendineux est vérifié à travers la plaie sans avoir besoin à l'agrandir. Cette technique permet d'avoir les avantages du traitement chirurgical et d’éviter les aléas de l'abord classique.

**Figure 1:**
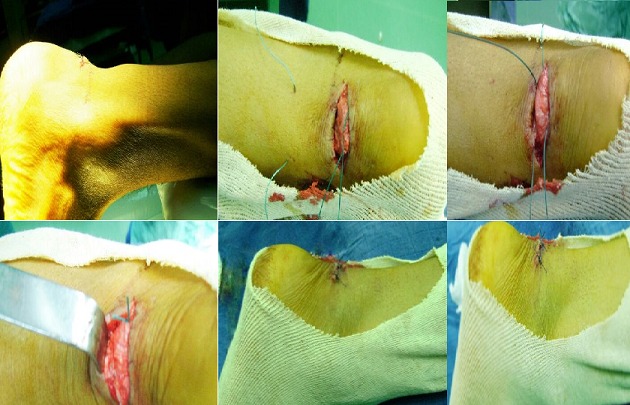
Les différents temps opératoires

